# Impaired trafficking and instability of mutant kidney anion exchanger 1 proteins associated with autosomal recessive distal renal tubular acidosis

**DOI:** 10.1186/s12920-022-01381-y

**Published:** 2022-10-31

**Authors:** Nipaporn Deejai, Nunghathai Sawasdee, Choochai Nettuwakul, Wanchai Wanachiwanawin, Suchai Sritippayawan, Pa-thai Yenchitsomanus, Nanyawan Rungroj

**Affiliations:** 1 Division of Molecular Medicine, Research Department, Faculty of Medicine Siriraj Hospital, Mahidol University, Bangkok, Thailand; 2 Division of Hematology, Department of Medicine, Faculty of Medicine Siriraj Hospital, Mahidol University, Bangkok, Thailand; 3 Division of Nephrology, Department of Medicine, Faculty of Medicine Siriraj Hospital, Mahidol University, Bangkok, Thailand; 4 Siriraj Genomics, Office of the Dean, Faculty of Medicine Siriraj Hospital, Mahidol University, Bangkok, Thailand

**Keywords:** *SLC4A1*, AE1, AR dRTA, Ovalocytosis, Hemolytic anemia

## Abstract

**Background:**

Mutations in *solute carrier family 4 member 1* (*SLC4A1*) encoding anion exchanger 1 (AE1) are the most common cause of autosomal recessive distal renal tubular acidosis (AR dRTA) in Southeast Asians. To explain the molecular mechanism of this disease with hematological abnormalities in an affected family, we conducted a genetic analysis of *SLC4A1* and studied wild-type and mutant AE1 proteins expressed in human embryonic kidney 293T (HEK293T) cells.

**Methods:**

*SLC4A1* mutations in the patient and family members were analyzed by molecular genetic techniques. Protein structure modeling was initially conducted to evaluate the effects of mutations on the three-dimensional structure of the AE1 protein. The mutant kidney anion exchanger 1 (kAE1) plasmid construct was created to study protein expression, localization, and stability in HEK293T cells.

**Results:**

We discovered that the patient who had AR dRTA coexisting with mild hemolytic anemia carried a novel compound heterozygous *SLC4A1* mutations containing c.1199_1225del (p.Ala400_Ala408del), resulting in Southeast Asian ovalocytosis (SAO), and c.1331C > A (p.Thr444Asn). Homologous modeling and in silico mutagenesis indicated that these two mutations affected the protein structure in the transmembrane regions of kAE1. We found the wild-type and mutant kAE1 T444N to be localized at the cell surface, whereas the mutants kAE1 SAO and SAO/T444N were intracellularly retained. The half-life of the kAE1 SAO, T444N, and SAO/T444N mutants was shorter than that of the wild-type protein.

**Conclusion:**

These results suggest impaired trafficking and instability of kAE1 SAO/T444N as the likely underlying molecular mechanism explaining the pathogenesis of the novel *SLC4A1* compound heterozygous mutation identified in this patient.

**Supplementary Information:**

The online version contains supplementary material available at 10.1186/s12920-022-01381-y.

## Background

Distal renal tubular acidosis (dRTA) is a rare genetic disorder (OMIM 611590) that is characterized by a failure of the distal nephron to secrete acid into the urine, which results in the development of metabolic acidosis [1]. The clinical symptoms of patients with dRTA include growth retardation, muscle weakness, hyperchloremic metabolic acidosis, osteomalacia, hypokalemia, and nephrocalcinosis or nephrolithiasis [2, 3]. Mutations of at least 5 human genes have been reported to cause hereditary dRTA. These genes include *SLC4A1*, *ATP6V1B1*, *ATP6V0A4*, *WDR72*, and *FOXI1*, encoding anion (Cl^−^/HCO_3_^−^) exchanger 1, B1-subunit of H^+^-adenosine triphosphatase (ATPase), a4-subunit of H^+^-ATPase, WD repeat-containing protein 72, and Forkhead box protein I1, respectively [4–8]. In addition, more recently, *ATP6V1C2* gene which encodes C2-subunit of the V-type proton ATPase has been reported as a potential cause of dRTA [9]. Among these reported genes, mutation of the *SLC4A1* gene was found in 70% of patients with dRTA in Thailand [10].

The human *SLC4A1* or *anion exchanger 1* (*AE1*) gene (Accession: NM_000342.4, OMIM + 109270) encodes both the erythroid anion exchanger protein in erythrocytes and the kidney anion exchanger 1 (kAE1) protein in α-intercalated cells of the kidney [11]. Thus, defects of the *SLC4A1* gene can cause morphological changes of red blood cells, including ovalocytosis and spherocytosis or dRTA condition [12]. Mutations in the *SLC4A1* gene have been found to cause both autosomal dominant and autosomal recessive (AR) forms of dRTA depending on the sites of the mutations. Our group previously reported the homozygous G701D mutation and the compound heterozygous G701D/S773P, G701D/A858D, Southeast Asian ovalocytosis (SAO, p.Ala400_Ala408del)/G701D and SAO/R602H mutation as the cause of AR dRTA in Thai patients [10, 13–15]. Previous population survey revealed the SAO mutation to be the most prevalent *SLC4A1* mutation in Southern Thai population, whereas the G701D mutation is the common *SLC4A1* mutation in Northeastern Thai population [16]. Moreover, hemolytic anemia was also found in patients with AR dRTA who carried the compound heterozygous SAO/G701D, SAO/Q759H, SAO/V850, and SAO/A858D mutations [10, 17, 18]. This data indicates that the SAO mutation combined with another mutation in the *SLC4A1* gene is likely to cause AR dRTA with hemolytic anemia.

During the process of attempting to identify *SLC4A1* mutations in a Thai female with both dRTA and mild hemolytic anemia, we discovered a novel compound heterozygous *SLC4A1* SAO/T444N mutation as the possible cause of the disease. To elucidate the underlying molecular mechanism(s) of the disease, the effects of this novel compound heterozygous *SLC4A1* mutation on the structure of kAE1 were predicted, and kAE1 protein expression and stability were examined in human embryonic kidney 293T (HEK293T) cells.

## Methods

### Subjects

The protocol for this study was approved by the Human Research Ethics Committee of the Siriraj Institutional Review Board, Faculty of Medicine Siriraj Hospital, Mahidol University, Bangkok, Thailand (COA no. Si 741/2013). Written informed consent was obtained from all study subjects.

A 35-year-old Thai female (hereafter referred to as ‘the patient’ or ‘our patient’) from Central Thailand who regularly attends medical service at the outpatient section of the Faculty of Medicine Siriraj Hospital, Mahidol University for evaluation and treatment of her dRTA and mild anemia conditions was recruited. Blood sample was prospectively collected from the patient for biochemical, hematological, and DNA analyses, and buccal cells were collected from her family members for DNA analysis.

### Mutation analysis

Genomic DNA samples from the patient and her family members were extracted from peripheral blood and buccal cells using a Puregene Blood Core Kit B (Qiagen, Hilden, Germany). Common *SLC4A1* SAO and G701D mutations screening [16] in the patient was performed using PCR and PCR–RFLP methods, as previously described [15]. The mutations in all exons of the *SLC4A1* gene were further analyzed using Sanger DNA sequencing. All twenty exons of the *SLC4A1* gene were amplified using twenty-two pairs of PCR primers (Additional file 1: Table S1) [13]. The PCR products were purified by using ExoSAP-IT™ Express PCR Product Cleanup Reagent (Thermo Fisher Scientific, Waltham, MA, USA). The purified PCR products were then analyzed by DNA sequencing (Bioneer, Daejeon, South Korea). The DNA sequencing results were compared with the reference nucleotide sequence of the *SLC4A1* gene (accession number GI: 171460929) using Clustal Omega program (EMBL-EBI, Wellcome Genome Campus, Cambridgeshire, England, UK). The possible disease-causing mutations identified in the patient were genotyped in the family members by PCR or PCR–RFLP methods. The T444N mutation was analyzed in all family members and in 200 normal control subjects by amplification of exon 12 of the *SLC4A1* gene. PCR products were digested with 3 U of *Pst*I (New England Biolabs, Ipswich, MA, USA) overnight at 37 °C and the digested PCR fragments were analyzed by 2% agarose gel electrophoresis.

### In silico analysis

The pathogenicity of the *SLC4A1* mutations identified in the patient was predicted using VarCards program [19]. The amino acid sequences of AE1 proteins from human and various vertebrate species were aligned using the Clustal Omega program.

### Protein structure modeling

The full-length AE1 structure [20] was used as a template to predict the three-dimensional structure of human wild-type and mutant kAE1 structures using the Swiss-model homology modeling server [21]. PyMOL 1.7.5.0 (DeLano Scientific LLC, Palo Alto, California, USA) was used to examine alterations in the protein structures and H-bond-forming patterns caused by amino acid changes as a result of the identified mutations.

### Site-directed mutagenesis

Four pcDNA3.1 plasmid constructs expressing kAE1 wild-type (WT) or kAE1 SAO fused with either Myc or HA (pcDNA3.1-kAE1 WT-Myc, pcDNA3.1-kAE1 SAO-Myc, pcDNA3.1-kAE1 WT-HA, and pcDNA3.1-kAE1 SAO-HA) were previously generated [22]. The kAE1-Myc protein has a Myc tag at position 557 in the third extracellular loop of the kAE1 protein, while the kAE1-HA protein has a HA tag at the C-terminus of the kAE1 protein. pcDNA3.1-kAE1 WT-Myc and pcDNA3.1-kAE1 WT-HA were used as templates for generation of pcDNA3.1-kAE1 T444N-Myc and pcDNA3.1-kAE1 T444N-HA by site-directed mutagenesis method using specific primers (*SLC4A1*-T444N forward: 5′-GGAGCTGCTGATCTCCAATGCAGTGCAGGGCATTC-3′; *SLC4A1*-T444N reverse: 5′-GAATGCCCTGCACTGCATTGGAGATCAGCAGCTCC-3′) and *Pfx* DNA polymerase (Invitrogen, Carlsbad, CA, USA), as described previously [22].

### Cell culture and transfection

The HEK293T cells were cultured in complete Dulbecco’s Modified Eagle’s Medium (Gibco; Thermo Fisher Scientific, Waltham, MA, USA) supplemented with 10% fetal bovine serum (Gibco; Thermo Fisher Scientific) and 1.2% penicillin G-streptomycin in an incubator at 37 °C with 5% CO_2_. HEK293T cells at 50–70% confluent growth were transiently transfected or co-transfected with 1 µg of recombinant plasmid per well in a 6 well plate using a Lipofectamine^®^ 2000 or Lipofectamine^®^ 3000 Transfection Kit (Invitrogen). Cells were harvested 48 h after transfection for further experiments. The HEK293T cells were transfected with one of the six plasmids (pcDNA3.1 plasmid expressing kAE1 WT, kAE1 SAO, or kAE1 T444N fused with either Myc or HA), or co-transfected with different pairs of plasmids containing wild-type or mutant kAE1-Myc tag and wild-type or mutant kAE1-HA tag for studies of expression, co-expression, cell surface expression, and cellular localization.

### Western blot analysis

The kAE1 proteins were examined by Western blot analysis, as previously described [22]. The wild-type and mutant kAE1 proteins were subjected to electrophoresis on 10% SDS-PAGE gel and transferred to nitrocellulose membranes. The membranes were incubated with mouse anti-Myc antibody (sc-40, Santa Cruz Biotechnology, Dallas, TX, USA) or mouse anti-GAPDH antibody (Santa Cruz Biotechnology) overnight at 4 °C. The membranes were then incubated with rabbit anti-mouse secondary antibody conjugated to horseradish peroxidase (Dako Cytomation, Glostrup, Denmark) for 1 h. The kAE1 proteins were detected by addition of SuperSignal West Pico Chemiluminescent Substrate (Thermo Fisher Scientific) according to the manufacturer’s protocol. The chemiluminescence signals were visualized using a G:BOX Chemiluminescence Imaging System, and their intensities were quantified using GeneTools software version 4.03 (Syngene, Cambridge, UK).

### Flow cytometry

The transfected cells were incubated with mouse anti-Myc antibody for 1 h on ice. The cells were further incubated with a goat anti-mouse Alexa 488-conjugated IgG secondary antibody (Invitrogen) for 30 min on ice in the dark. The cells were analyzed using a BD Accuri™ C6 Plus Flow Cytometer (BD Biosciences, San Jose, CA, USA).

### Immunofluorescence assay

To examine cellular localization of the wild-type and mutant kAE1 proteins, glass cover slips were coated with 100 µg/ml poly-D-lysine for 5 min and then washed with sterile water. After the cover slips were dried for at least 2 h, HEK293T cells were grown on the cover slips in a 6-well plate. The transfected cells were fixed, permeabilized, and incubated with primary and secondary antibodies, as described above. The nuclei of cells were stained with Hoechst 33342 (Molecular Probes, Eugene, USA). After washing, the cover slips were mounted with 50% glycerol in 1x PBS. The localization of kAE1 proteins was examined using a LSM 800 Confocal Microscope (Carl Zeiss Microscopy, Jena, Germany).

### Co-immunoprecipitation (Co-IP)

HEK293T cells were separately co-transfected with different pairs of plasmid constructs containing wild-type kAE1-Myc tag or mutant kAE1-Myc tag, and wild-type kAE1-HA tag or mutant kAE1-HA tag. The cell lysates were incubated with mouse anti-HA antibody and protein G-Sepharose beads. After washing steps, the bound proteins were eluted and HA-tagged proteins were detected by SDS-PAGE and Western blot analysis with mouse anti-Myc antibody or mouse anti-HA antibody. After washing, the membranes were incubated with rabbit anti-mouse secondary antibody conjugated to horseradish peroxidase and protein bands were detected as previously described.

### Investigation of protein stability by using cycloheximide

Twenty-four hours after transfection, new protein synthesis was inhibited with 100 μg/ml of cycloheximide (CHX), which is a protein synthesis inhibitor [23]. Cells were collected at 0, 6, 12, 24, and 36 h after CHX treatment, and the stability of the kAE1 proteins was examined by Western blot analysis, as previously described [22].

### Statistical analysis

All experiments were conducted at least three times to obtain the datasets for calculation of the mean ± standard error of the mean. One-way ANOVA followed by Tukey’s multiple comparisons test was used for statistically significant differences between the means of two groups. A *p *value < 0.05 was considered statistically significant for all tests.

## Results

### Patient and clinical study

**Fig. 1:**
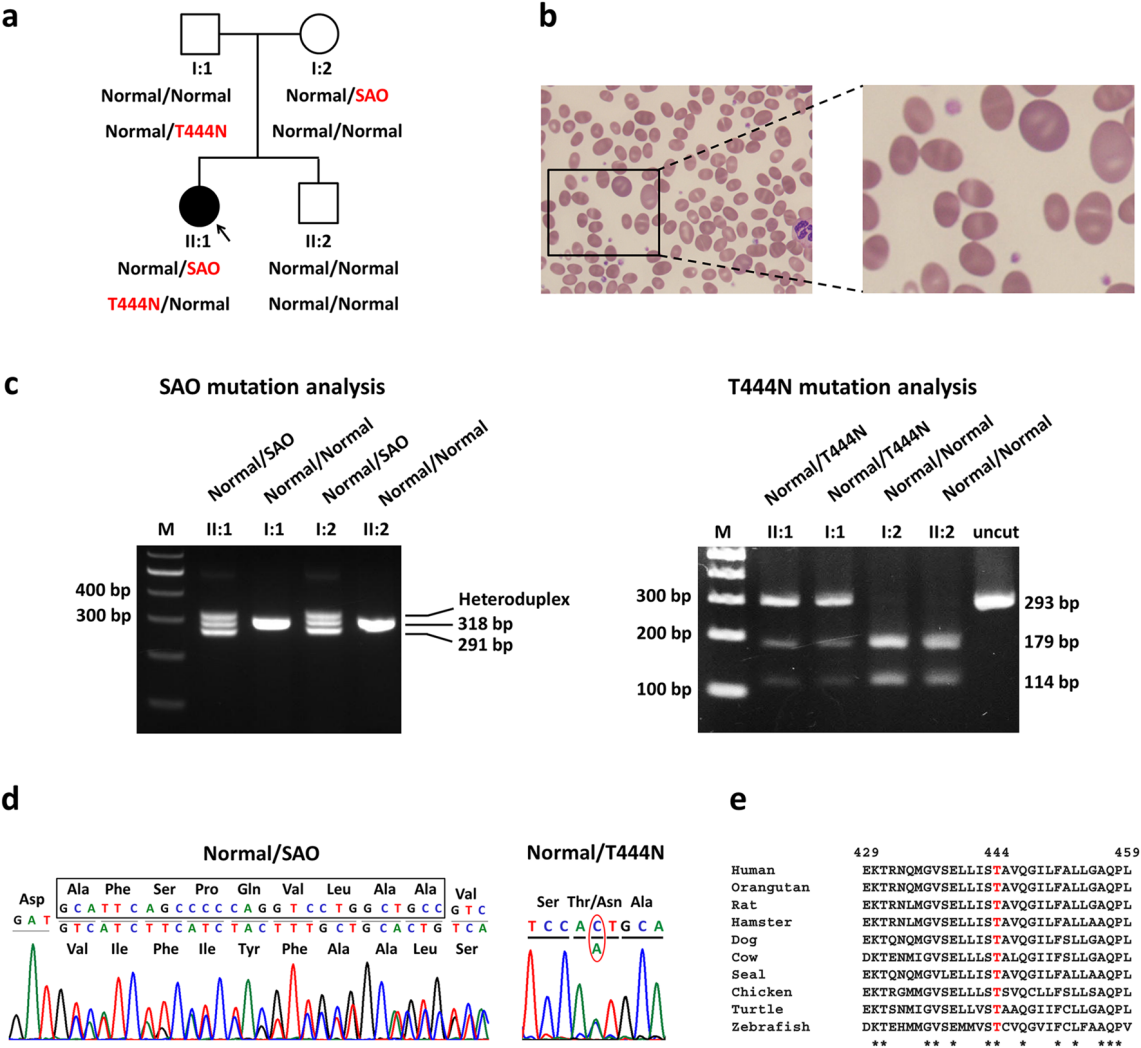
Segregation analysis of the *SLCA41* SAO/T444N mutation in the affected family. **a** Pedigree and segregation of SAO and T444N mutations in a family affected by dRTA. **b** Red blood cell morphology of the patient showing ovalocytes, theta cells, and stomatocytes. **c** SAO mutation screening using PCR technique and T444N mutation analysis by the PCR–RFLP method. **d** Sequence analysis of the SAO and T444N mutations in the patient showing a 27-nucleotide deletion in codons 400 to 408, which corresponds with the heterozygous SAO mutation, and a base substitution leading to a missense (threonine to asparagine) mutation at codon 444 of the protein. **e** Multiple amino acid sequence alignment of the AE1 protein among ten vertebrate species in the region where the T444N mutation was identified. Full-length gels are presented in Additional file 1: Fig. S1

A family with one patient and three family members was recruited for this study (Fig. 1a). Laboratory investigations showed that the patient had persistent hyperchloremic metabolic acidosis, with urine pH of 7.0, a blood HCO_3_^−^ level of 20 mEq/L, and creatinine clearance of 70.6 cc/min/1.73 m^2^—all of which correspond with the diagnostic criteria for dRTA. Morphological examination of the peripheral blood smear showed the presence of ovalocytes, theta cells (red cells with transverse ridges), and stomatocytes (Fig. 1b). Findings from the hematological study (Additional file 1: Table S2) indicated that the patient had mild hemolytic anemia from hereditary ovalocytosis.

### Identification of mutations in the *SLC4A1* gene

The *SLC4A1* SAO and G701D mutations were screened in the patient by PCR and PCR–RFLP methods, respectively. The results showed that the patient (II:1) had three bands, including a heteroduplex DNA (> 318 bp), 318 bp and 291 bp, which indicated that she carries the heterozygous of SAO mutation. She inherited this mutation from her mother (I:2) who was found to have the same DNA pattern. In contrast, her father (I:1) and younger brother (II:2) had only one band of 318 bp, indicating the absence of the SAO mutation (Fig. 1c) (Additional file 1: Fig. S1). The result of c.2102G > A (p.Gly701Asp) mutation screening showed the absence of this mutation in the patient and all other family members (data not shown). Due to the absence of common G701D mutation in this family, we decided to analyze all exons and exon–intron boundaries of the *SLC4A1* gene using Sanger DNA sequencing. The results revealed that the patient had a novel compound heterozygous SAO/T444N mutation (Fig. 1d).

The T444N mutation was then analyzed in all family members and in 100 normal control subjects from Songkla Province in Southern Thailand, and 100 normal control subjects from Bangkok in Central Thailand by PCR–RFLP method to generate 3 patterns that represented the following 3 different genotypes: homozygous wild-type (CC; 179 and 114 bp), heterozygous T444N (CA; 293, 179, and 114 bp), and homozygous T444N (AA; 293 bp). The result indicated that the patient (II:1) and her father (I:1) had the heterozygous T444N, and her mother (I:2) and younger brother (II:2) had the homozygous wild-type genotype (Fig. 1c) (Additional file 1: Fig. S1). The result of genotype analysis revealed no T444N mutation in any of the 200 DNA samples from the normal control subjects.

### Bioinformatic analysis of mutation in the *SLC4A1* gene

Bioinformatic analysis of the T444N using VarCards revealed that 17 of 21 algorithms showed a deleterious effect of this mutation (Additional file 1: Table S3). Multiple amino acid sequence alignment of the human AE1 protein with different vertebrate species showed T444 to be a highly conserved amino acid residue in the vertebrate evolution from zebrafish to human species (Fig. 1e).

### Structural modeling of the wild-type and mutant kAE1 proteins

**Fig. 2:**
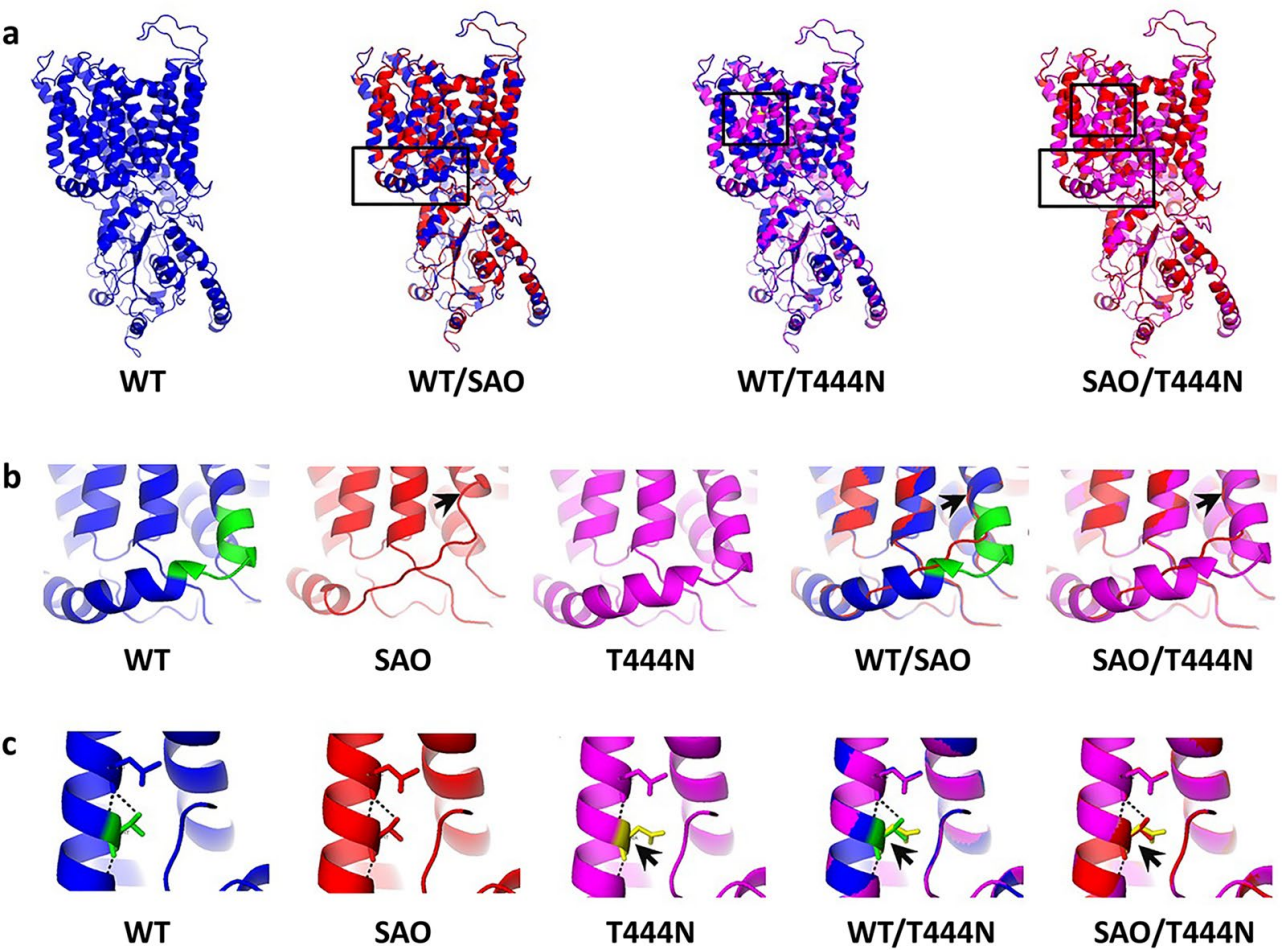
Three-dimensional structure of the wild-type (WT) and mutant kAE1 proteins. **a** Three-dimensional structure of wild-type kAE1 (blue) and superimposed structures of WT/SAO (blue/red), WT/T444N (blue/magenta), and SAO/T444N (red/magenta) as predicted by Swiss-model homology modeling server. **b** The protein structure where the SAO alteration is located. The deletion of residues 400–408 (green), which causes SAO, adversely affects the protein structure deletion and uncoiling of the helix (indicated by arrows). **c** The protein structure where the T444N alteration is located (indicated by arrows). There are two putative H-bonds connecting T444 (green) to L440 compared to only one putative H-bond connecting N444 (yellow) to L440. The dashed lines indicate the predicted H-bonds between residues

The identified SAO and T444N alterations were found to affect the protein structure in the transmembrane regions of the kAE1 protein (Fig. 2a). The deletion of residues 400–408, which causes SAO, was found to affect the protein structure by deletion and uncoiling of the helix (Fig. 2b). The T444N alteration was found to affect the protein structure by alterations in H-bond forming patterns (Fig. 2c).

### Wild-type and mutant kAE1 protein expression by western blot analysis

**Fig. 3:**
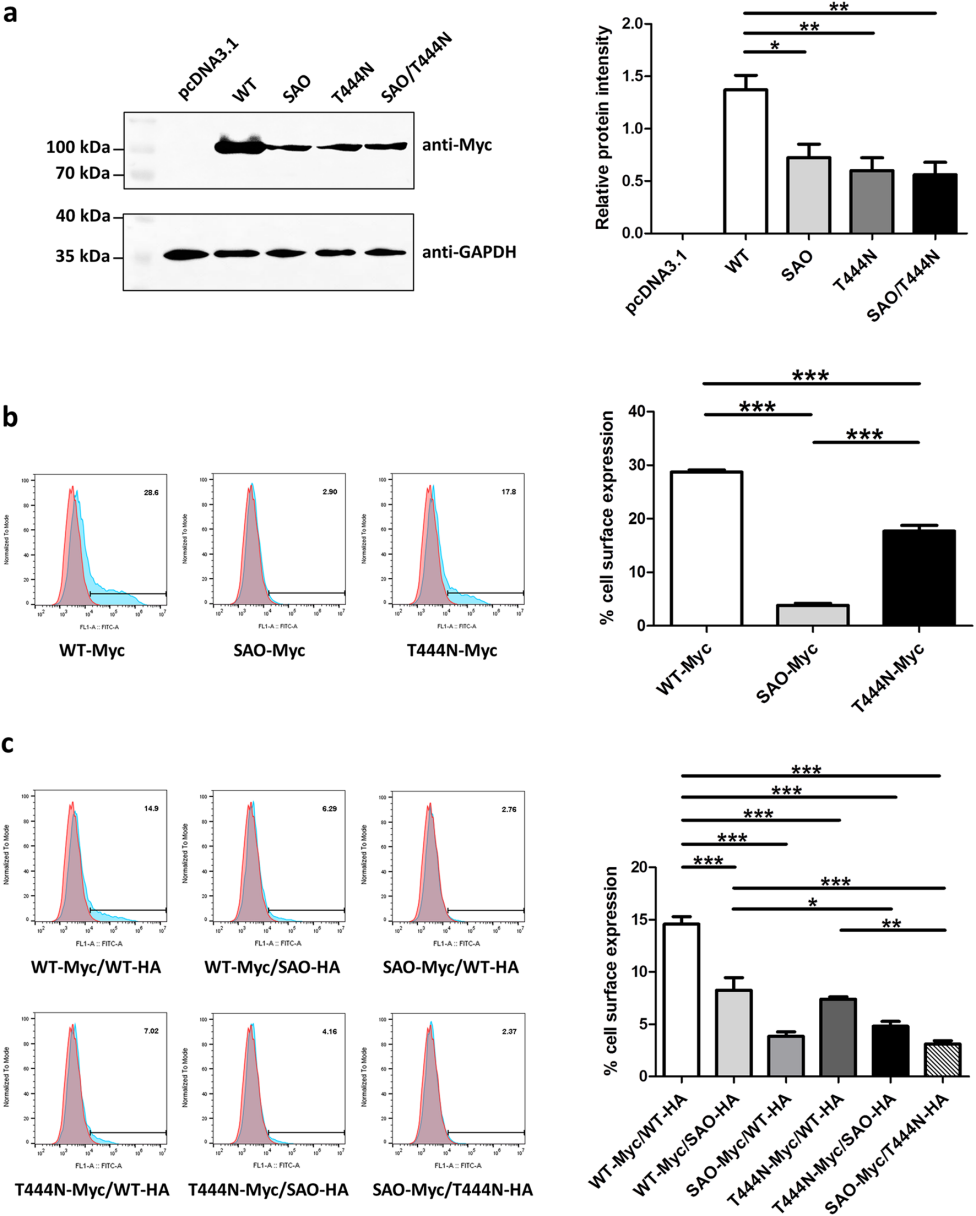
Expression of the wild-type and mutant kAE1 proteins. **a** Western blot analysis of the wild-type and mutant kAE1 proteins and endogenously expressed GAPDH in HEK293T cells. Images of the blots cropped from different parts of the same blot were separated by dividing lines. Full-length blots are presented in Additional file 1: Fig. S2. **b** Analysis of cell surface expression of the wild-type and the mutant kAE1 SAO and T444N proteins by flow cytometry. **c** Analysis of cell surface expression in co-expressions of the wild-type and the mutant kAE1 SAO or T444N proteins or the mutant kAE1 SAO and T444N proteins in HEK293T cells by flow cytometry. The transfected cells were stained with mouse anti-Myc antibody, followed by AlexaFluor 488-conjugated anti-mouse antibody. Statistical analysis was performed using one-way ANOVA followed by Tukey’s multiple comparisons test (*p < 0.05, **p < 0.01, ***p < 0.001)

The expression levels of mutant kAE1 SAO, T444N, and SAO/T444N proteins were found to be significantly decreased, and to be approximately 40% lower than that of the wild-type kAE1 protein (Fig. 3a) (Additional file 1: Fig. S2).

### Cell surface expression of the wild-type and mutant kAE1 proteins by flow cytometry

The expression of wild-type and mutant kAE1-Myc protein on the cell surface was examined by flow cytometry using mouse anti-Myc antibody. The result showed the cell surface expressions of the kAE1 SAO-Myc (3.80% ± 0.42%) and kAE1 T444N-Myc (17.68% ± 1.06%) were significantly different when compared with that of kAE1 WT-Myc (28.70% ± 0.41%) (Fig. 3b).

The cell surface expressions of kAE1 WT-Myc co-expressed with kAE1 SAO-HA (8.25% ± 1.21%), kAE1 SAO-Myc co-expressed with kAE1 WT-HA (3.84% ± 0.44%), and kAE1 T444N-Myc co-expressed with kAE1 WT-HA (7.41% ± 0.21%) were significantly lower than that of kAE1 WT-Myc co-expressed with kAE1 WT-HA (14.58% ± 0.72%). Furthermore, the co-expression of kAE1 T444N-Myc with kAE1 SAO-HA and kAE1 SAO-Myc with kAE1 T444N-HA in HEK293T cells, to mimic the compound heterozygous mutation observed in our patient, indicated that cell surface expression of these mutants (4.82% ± 0.45% and 3.11% ± 0.31%) were low and significantly different when compared to that of kAE1 WT-Myc co-expressed with kAE1 WT-HA or kAE1 SAO-HA, and kAE1 T444N-Myc co-expressed with kAE1 WT-HA (Fig. 3c).

### Cellular localization of the wild-type and mutant kAE1 proteins by immunofluorescence staining

The transfected HEK293T cells expressing either kAE1 WT-Myc or kAE1 WT-HA showed their expressions at the cell surface, and a similar finding was observed in the transfected cells expressing the kAE1 T444N-Myc. In contrast, the transfected cells expressing either kAE1 SAO-Myc or kAE1 SAO-HA demonstrated predominant intracellular retention of the kAE1 protein (Additional file 1: Fig. S3).

**Fig. 4:**
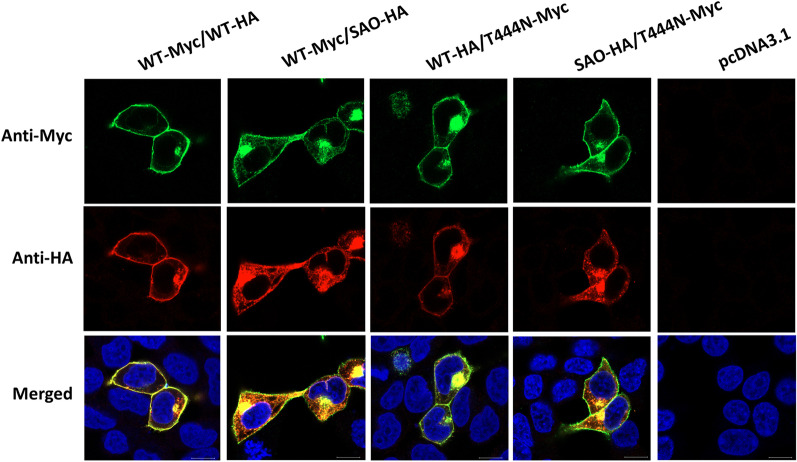
Cellular localization of the wild-type and mutant kAE1 SAO or T444N proteins co-expressed in different conditions in HEK293T cells as detected by immunofluorescence assay. The transfected cells were stained with mouse anti-Myc and rabbit anti-HA antibodies, followed by AlexaFluor 488-conjugated anti-mouse antibodies (green), AlexaFluor 594-conjugated anti-rabbit antibodies (red), and Hoechst 33342 (blue). Scale bars: 10 µm

The co-expression of kAE1 WT-Myc with kAE1 WT-HA showed obvious cell surface expression, while the co-expression of kAE1 WT-HA and kAE1 T444N-Myc showed expression predominantly on the cell surface, with some intracellular localization. To mimic SAO/T444N compound heterozygous condition, kAE1 SAO-HA and kAE1 T444N-Myc were co-expressed in HEK293T cells. The result showed predominant retention of the kAE1 proteins in the cytoplasm, while kAE1 T444N-Myc protein was still abundant on the cell surface. These results indicate that T444N-Myc tagged proteins do not seem to be affected by WT or SAO HA tagged proteins. A similar result was also obtained from the co-expression of kAE1 WT-Myc and kAE1 SAO-HA (Fig. 4).

### Detection of wild-type and mutant kAE1 protein interactions by co-immunoprecipitation

**Fig. 5:**
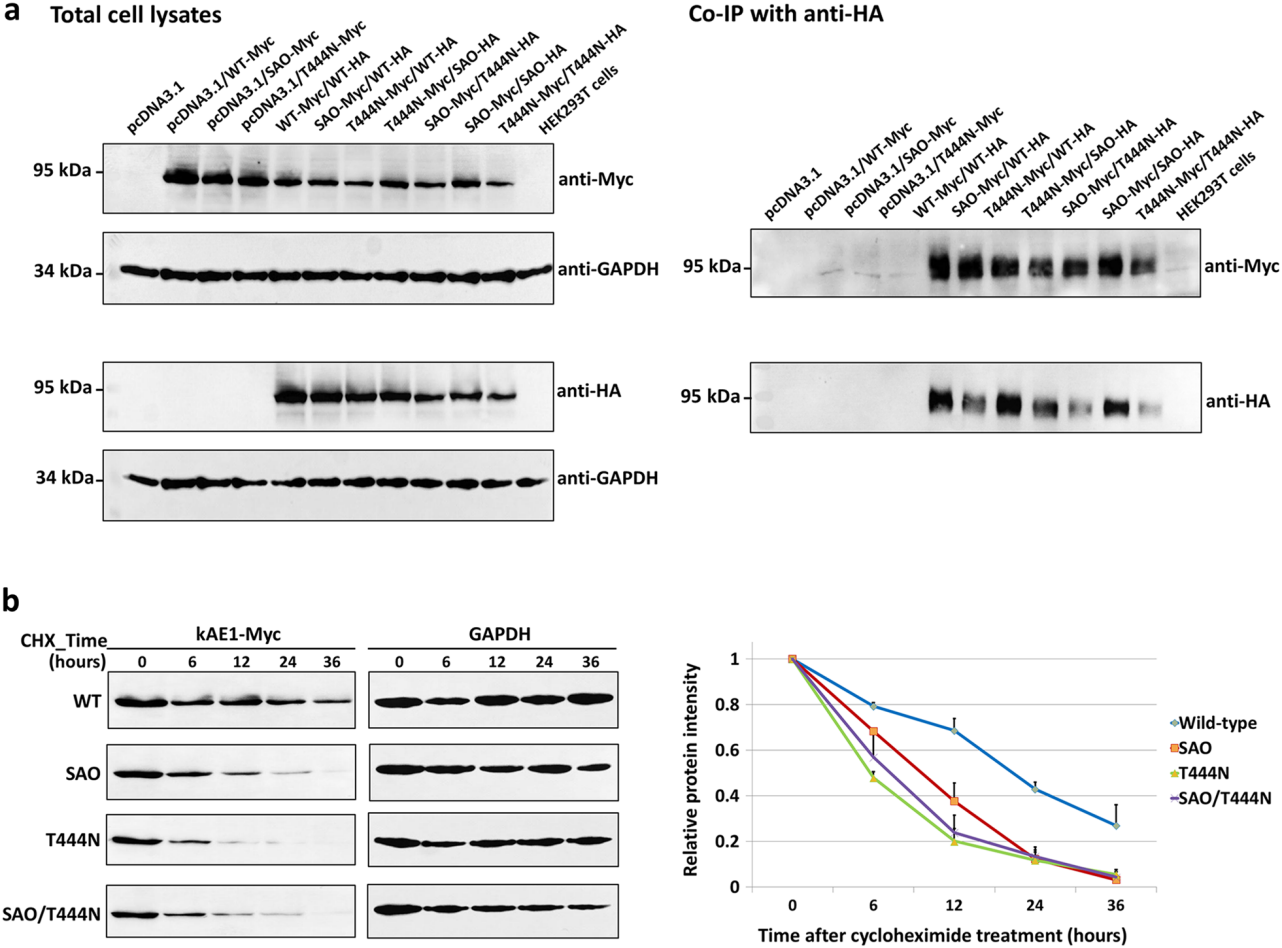
Interactions and stability of the wild-type and mutant kAE1 proteins. **a** Interactions between wild-type kAE1 and mutant kAE1 SAO or kAE1 T444N, and mutant kAE1 SAO and kAE1 T444N co-expressed in HEK293T cells were examined by the co-immunoprecipitation (Co-IP) method. Images of the blots cropped from different parts of the same blot, or from different blots were separated by dividing lines. Full-length blots are presented in Additional file 1: Fig. S4. **b** Stability of wild-type and mutant kAE1 proteins in transfected HEK293T cells after treatment with CHX and detection by Western blot analysis at 0, 6, 12, 24, and 36 h. Images of the blots cropped from different parts of the same blot, or from different blots were separated by dividing lines. Full-length blots are presented in Additional file 1: Fig. S5

We also confirmed the interactions between the wild-type and mutant kAE1 proteins by co-immunoprecipitation (Co-IP) method (Fig. 5a) (Additional file 1: Fig. S4). The results showed that kAE1 T444N-Myc protein could interact with either kAE1 WT-HA, kAE1 SAO-HA or kAE1 T444N-HA. Furthermore, the interactions could also be observed in the co-expressions of kAE1 WT-Myc and kAE1 WT-HA, kAE1 SAO-Myc and kAE1 WT-HA, kAE1 SAO-Myc and kAE1 SAO-HA, and kAE1 SAO-Myc and kAE1 T444N-HA.

### Stability of wild-type and mutant kAE1 proteins expressed in HEK293T cells

The results showed the level of the wild-type kAE1 protein to be initially constant, and then reduced by 50% after CHX treatment for more than 18 h. In contrast, the levels of all mutant kAE1 proteins were more rapidly reduced compared to that of the wild-type kAE1 protein. The half-life of kAE1 SAO protein was approximately 9 h. In contrast, the half-life in the kAE1 T444N and SAO/T444N proteins was approximately 6 h for each (Fig. 5b) (Additional file 1: Fig. S5).

## Discussion

Mutations in the human *SLC4A1*, *ATP6V1B1*, *ATP6V0A4*, *WDR72*, *FOXI1*, and *ATP6V1C2* genes were previously reported to be causes of dRTA [4–9]. Among these 6 reported genes, mutations in the *SLC4A1* gene are the most common cause of AD and AR dRTA, which are conditions that have been intensively studied in Thai population [10, 24–26]. In in vivo studies, a mouse model that lacks the *Ae1* gene exhibited dRTA, while mice carrying heterozygous for the *Ae1*-deficient allele had no apparent defect [27]. Recently, Mumtaz et al*.* [28] reported that they generated an *Ae1* R607H knock-in mouse, which corresponds to the R589H mutation of the *SLC4A1* gene that is a common cause of autosomal dominant dRTA. The heterozygous and homozygous R607H knock-in mice have incomplete dRTA without red blood cell abnormalities. The expression level of *Ae1* in type A-intercalated cells (A-ICs) in mutant mice was decreased, but basolateral targeting of the mutant protein was preserved [28].

In the present study, we discovered a novel compound heterozygous *SLC4A1* SAO/T444N mutation in a Thai female with dRTA and mild hemolytic anemia. The SAO mutation is a common *SLC4A1* mutation that causes AR dRTA in patients from Southern Thailand [16]. However, the heterozygous SAO mutation alone does not cause AR dRTA [13, 17], and the homozygous SAO mutation has not been identified in dRTA patients. The explanation for this absence of the homozygous SAO mutation was that this condition may be lethal [29]. In several previous reports, the SAO mutation was observed in Thai patients with AR dRTA in compound heterozygotes with G701D [10, 13] or R602H mutations [14]. Hemolytic anemia was reported to be most commonly found in patients who carried the compound heterozygous SAO/G701D mutation [10]. Interestingly, hemolytic anemia was also found in patients with AR dRTA from Malaysia and Papua New Guinea who had a compound heterozygous mutation of SAO and G701D, Q759H, V850 or A858D in the *SLC4A1* gene [17, 18]. Thus, a compound heterozygous mutation comprising mutations of both SAO and another mutation in the *SLC4A1* gene is likely to cause AR dRTA with hemolytic anemia. Although the T444N mutation was first identified in our patient, the same variant was reported in the gnomAD database at an extremely low frequency (rs754973425; allele count 7/250734; allele frequency 0.00002792). The result of bioinformatics analysis showed the T444N mutation to be predicted to be disease-causing and the T444 was found to be evolutionarily conserved in vertebrates, which supports its functional and structural significance. The SAO and T444N mutations were also predicted to affect the protein structure by deletion and uncoiling of the helix and alterations in H-bond forming patterns, respectively. These data strongly suggest this novel compound heterozygous *SLC4A1* mutation as being the cause of dRTA and mild hemolytic anemia in this patient.

The underlying molecular mechanism of dRTA caused by this novel compound heterozygous *SLC4A1* mutation was investigated by in vitro studies of the mutant kAE1 protein in HEK293T cells. The rationale for performing this study in HEK293T cells is to rapidly investigate the expression, stability, and localization of kAE1 proteins in non-polarizable embryonic kidney cells. We found the expressions of the mutant kAE1 proteins (SAO, T444N, and SAO/T444N) to be significantly reduced compared to that of the wild-type kAE1 protein in Western blot analysis. The most sensible explanation of this observation is that these mutant AE1 proteins were unstable when they were expressed in HEK293T cells, as shown in the protein stability study using CHX treatment. These results are partly consistent with the result of the previous study that reported the expression level and stability of the mutant kAE1 SAO protein to be significantly decreased [30].

The cell surface expression and cellular localization of the wild-type and mutant kAE1 proteins in HEK293T cells were investigated by flow cytometry and immunofluorescence staining, respectively. The expression of wild-type and mutant kAE1-Myc proteins on the cell surface could be examined by flow cytometry, because Myc epitope was inserted at position 557 in the third extracellular loop of kAE1 protein. However, the expression of the kAE1-HA protein on the cell surface could not be detected by the anti-HA antibody staining and flow cytometry analysis because the HA epitope was tagged at the C-terminus of kAE1, which was intracellularly located. When individually expressed, the wild-type kAE1 and mutant kAE1 T444N proteins showed their expressions at the cell surface, whereas the mutant kAE1 SAO protein had intracellular retention, which is likely due to a failure in its trafficking to locate at the cell surface, as previously reported [22, 31]. To mimic heterozygous *SLC4A1* SAO, heterozygous T444N, and compound heterozygous SAO/T444N conditions in the mother, father, and patient, respectively, kAE1 WT/SAO, WT/T444N, and SAO/T444N were co-expressed and examined in HEK293T cells. The results showed that the wild-type kAE1 protein could partially rescue the kAE1 SAO protein to express at the cell surface, as previously noted [30]. This effect was previously described as the ‘dominant positive effect’ of the wild-type kAE1 to the mutant kAE1 protein [12]. Moreover, the kAE1 WT and kAE1 T444N were mainly co-localized at the cell surface with little intracellular retention of both kAE1 proteins. Our results showed no impaired trafficking in the wild-type kAE1 protein co-expressed with the mutant kAE1 SAO or T444N protein, which may explain the absence of dRTA in the patient’s parents with heterozygous SAO or T444N mutation.

The observed co-localization of kAE1 SAO with kAE1 T444N showed predominant retention of the kAE1 protein (kAE1 SAO-HA) in the cytoplasm with some cell surface expression of kAE1 T444N-Myc protein. This result indicates that the kAE1 T444N protein did not assist the kAE1 SAO protein to locate at the cell surface. This is also supported by the results of flow cytometry analysis that when kAE1 SAO-Myc was co-expressed with either kAE1 WT or kAE1 T444N in HEK293T cells, kAE1 SAO was intracellularly retained and not rescued to the cell surface. The interactions between the wild-type and mutant kAE1 proteins were confirmed by Co-IP method. The results showed that the wild-type and mutant kAE1 proteins can physically interact to each other (Fig. 5a). These results confirm those that have previously been reported [22], and also indicate that SAO and T444N mutations in the *SLC4A1* gene do not affect the structural folding involving in the interacting property of kAE1 protein.

In the patient with SAO/T444N mutations, heterodimer of kAE1 SAO and kAE1 T444N proteins (kAE1 SAO/T444N), homodimers of kAE1 SAO (kAE1 SAO/SAO) and kAE1 T444N (kAE1 T444N/T444N) proteins were likely to form. Both heterodimer of kAE1 SAO/T444N and homodimer of kAE1 SAO/SAO proteins were intracellularly retained and unstable, which were targeted for degradation. The deletion or missense mutations in the protein structure could be recognized by cellular quality control with subsequent targeting of the protein degradation mechanism [32, 33]. Only the homodimer of kAE1 T444N/T444N proteins would be able to traffic to the cell surface. Given that kAE1 T444N was present at the cell surface to the same extent as kAE1 WT, if it is functional and able to physically interact with kAE1 SAO, one would expect a phenotype similar to heterozygous SAO individuals with one functional allele. However, given that this patient has dRTA, this suggests that the function of kAE1 T444N is impaired. The resulting rapid degradation limits the amount of heterodimer and homodimer formation in these mutant kAE1 proteins, which leads to low levels of kAE1 T444N trafficking to the cell surface to maintain normally physiological function in acid–base regulation—all of which resulted in the development of dRTA in this patient. However, a functional assay in polarizable and epithelial kidney cells, such as MDCK cells, should be performed to investigate the localization and function of the kAE1 T444N protein, which could more completely elucidate the molecular mechanism of dRTA associated with T444N mutation.

## Conclusions

This is the first report of a Thai patient with AR dRTA and mild hemolytic anemia caused by a novel compound heterozygous *SLC4A1* SAO/T444N mutation. Instability in both of the mutant kAE1 SAO and T444N proteins resulting in low levels of expression and reduced trafficking to the cell surface to maintain normal acid–base balance is the most likely underlying molecular mechanism of dRTA in the patient reported in this study.

## Supplementary Information

**Additional file 1: Table S1:** PCR primers for amplification of all exons and exon-intron boundaries of the *SLC4A1* gene. **Table S2:** Clinical and laboratory data of patient (II:1). **Table S3:** Results of bioinformatic analyses of the T444N mutation using VarCards web-based program. **Fig. S1:** The original and unprocessed versions of Fig. 1c. **Fig. S2:** The original and unprocessed versions of Fig. 3a. **Fig. S3:** Cellular localization of wild-type and mutant kAE1 proteins in HEK293T cells detected by immunofluorescence assay. **Fig. S4:** The original blot and unprocessed versions of Fig. 5a. **Fig. S5:** The original blot and unprocessed versions of Fig. 5b.

## Data Availability

All data generated or analysed during this study are included in this published article and its supplementary information files. The T444N variant has been submitted to ClinVar database (accession number: SCV002522620).

## Abbreviations

SLC4A1: Solute carrier family 4 member 1
AE1: Anion exchanger 1
AR: Autosomal recessive
dRTA: Distal renal tubular acidosis
SAO: Southeast Asian ovalocytosis
kAE1: Kidney anion exchanger 1
CHX: Cycloheximide
