# Supplementary material for: Impaired trafficking and instability of mutant kidney anion exchanger 1 proteins associated with autosomal recessive distal renal tubular acidosis
Source: BMC Med Genomics. 2022 Oct 31;15:228. doi: 10.1186/s12920-022-01381-y (PMC9623938; doi:10.1186/s12920-022-01381-y)

Table S1 PCR primers for amplification of all exons and exon-intron boundaries of the *SLC4A1* gene.

Location	Primer name	Primer sequence	Annealing temperature (°C)	PCR product (bp)
Promoter	In31L	5′-CAGTTTGGGACAAGGGCGTG-3′	69	770
	In32R	5′-CGGTGTCGTGAGCTGAAAACC-3′		
Exon 1*	1L1	5′-GTGGGGCGGGCAGATTAG-3′	65	199
	1R1	5′-AGGCACCGAAGACCATCCC-3′		
Exon 2*	2L	5′-CCTCTGGAGGAGGACCAG-3′	60	232
	2R	5′-ACCCCCAACGAAGATAGG-3′		
Exon 3*	3L	5′-CTTCGTTGGGGGTGGAAGAG-3′	60	235
	3R	5′-GTGGAGAAGGGGAGAGGACAA-3′		
Exon 4	4L	5′-GTCTCTGAGGCTCACAGTGGATG-3′	63	226
	4R	5′-ATCCCCTTGCTCCTCTCTTCC-3′		
Exon 5	5L	5′-TGAGCACCCACTATGCCCT-3′	63	299
	5R	5′-CAGCACCCCACAACAATCCTC-3′		
Exon 6	6L	5′-AGATGAGGATTGTTGTGGGGT-3′	63	261
	6R	5′-CAAGTGGGCTGGGGAAGTG-3′		
Exon 7	7L	5′-CACCACTGATAGCTCAGCCTG-3′	60	243
	7R	5′-TGAGAAAGCTCTCTCCTTGCCC-3′		
Exon 8	8L	5′-GAGAATGGGAAGGGGAGGATG-3′	60	244
	8R	5′-GGTCCAGGCTGAGGGAAAGAC-3′		
Exon 9	9L	5′-TCTTCAGCACACCCACCCTG-3′	60	299
	9R	5′-TCAGCCACCATGCAGGTCC-3′		
Exon 10*	10L	5′-TCCTGGCAATGGGAGCTG-3′	65	483
	10R	5′-GATTGTCTGATGGGAATGGGG-3′		
Exon 11	11L	5′-CCTCACCTCCTCCAGCTACTCC-3′	67	318
	11R	5′-CAGAAGTTGGGGCTGAGACAGAG-3′		
Exon 12	12L	5′-GCTCTATGGGCTCCTGGAAAT-3′	62	293
	12R	5′-AAAGGGTCTTGGGGCAAGG-3′		
Exon 13	13L	5′-CTGTCATGTCCCCCGCAC-3′	58	339
	13R	5′-TGTCTCAGTCTTATACACAACCTCC-3′		
Exon 14	14L	5′-TGGTGGTATTTTCCAGCCCAAG -3′	60	320
	14R	5′-GCACTGAGGAATTTGGAGCGG-3′		
Exon 15	15L	5′-AAGGCAGGAGGTGGGGAGTGACTG-3′	70	201
	15R	5′-GGAAATGAGGACCTGGGGGGTATC-3′		
Exon 16	16L	5′-TCCTGCTCCCACCCTTCCCC-3′	68	276
	16R	5′-TCTGCCTCCCACCCTCCCAG-3′		
Exon 17	17L	5′-TGGAGGAGGCAGGGGAGAAC-3′	67	347
	17R	5′-GGGGCAGGAGGATGGTGAAG-3′		
Exon 18	18L	5′-ATATGGTGCCTGTGTTTTATTCCC-3′	65	336
	18R	5′-TGCCTATCACACCCCAGCAC-3′		
Exon 19	19L	5′-GGTACAGGACCCTTTTCTGG-3′	60	334
	19R	5′-GCCTGCCCTAGTTCTGAGAC-3′		
Exon 20*	20L	5′-CAAGACAGCCAGACCCTCTC-3′	61	335
	20R	5′-CTTCCTTCCCCACCCACAG-3′		
PolyA*	PolAL	5′-GATGTCCCTGTCTCCCCCTC-3′	60	201
	PolAR	5′-CCTAGTTCAGCCAAGACCC-3′		

PCR polymerase chain reaction, *bp* base pairs

* PCR primers were newly designed in this study

Table S2 Clinical and laboratory data of patient (II:1).

Patient data	II:1	Normal range
**Clinical chemistry**		
Urine pH[a]	7	4.6-8
Serum Bicarbonate [a] (mEq/L)	20	22-29
Potassium (K^+) (mmol/L)	3.5	3.4-4.5
BUN (mg/dl)	9.5	6-20
Creatinine (mg/dl)	0.93	0.51-0.95
Creatinine Clearance[a] (cc/minute/1.73 m^2)	70.6	< 60
**Hematology**		
Hemoglobin[b] (g/dL)	11.7	12.5-16.8
Hematocrit[b] (%)	31.7	37.1-42.7
Reticulocyte count[b] (%)	3.2	0.7-2.3
Absolute count	0.101	0.023-0.099
RBC (x 10^6/µl)	3.17	3.90-6.01
MCV[b] (fL)	100	82-97
MCH (pg)	37	27-31
MCHC[b] (g/dL)	37	32-36
RDW[b] (%)	24.5	12.5-14.9
White blood cell count (x 10^3/µl)	11.5	5.3-10
Neutrophils (%)	67.8	59-69
Lymphocyte (%)	23.4	34-42
Monocyte (%)	4.3	4-8
Eosinophils (%)	3.7	0-5
Basophils (%)	0.8	0-1
Platelet count (x 10^3/µl)	257	157-420

BUN Blood urea nitrogen, *RBC* Red blood cell, *MCV* Mean corpuscular volume, *MCH* Mean corpuscular hemoglobin, *MCHC* Mean corpuscular hemoglobin concentration, *RDW* Red cell distribution width

[a] Diagnostic criteria for patient with dRTA

[b] Diagnostic criteria for patient with anemia

Fig. S1 The original and unprocessed versions of Fig. 1c.

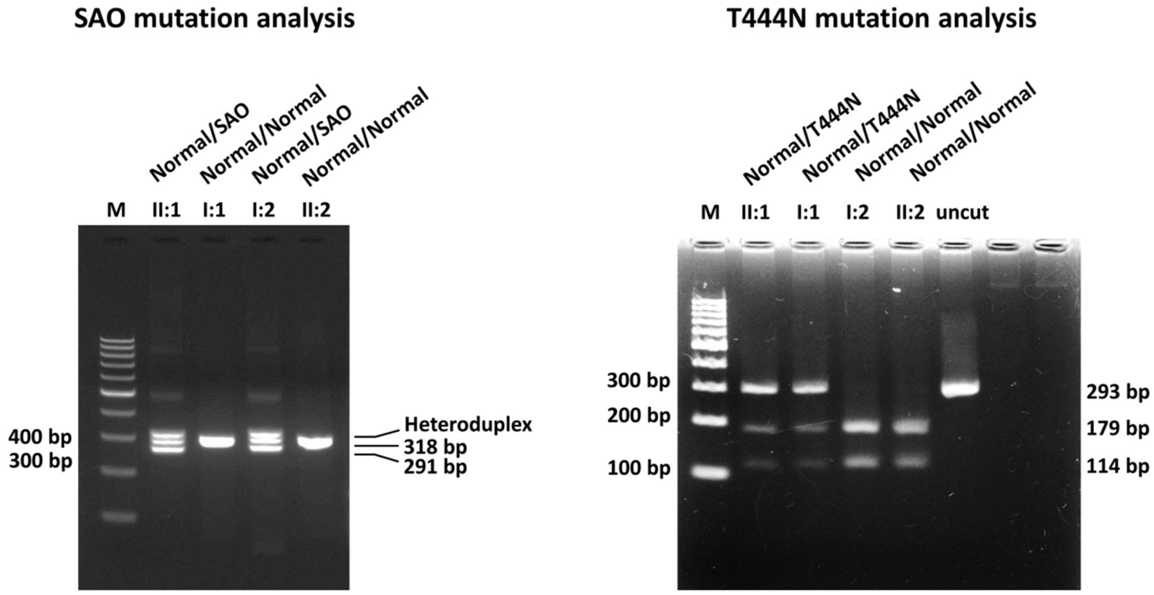

Table S3 Results of bioinformatic analyses of the T444N mutation using VarCards web-based program.

Algorithm	Score	Prediction
SIFT	0.004	Damaging
Polyphen-2_HDIV	1	Probably damaging
Polyphen-2_HVAR	0.999	Probably damaging
LRT	0	Deleterious
MutationTaster	1.0	Disease causing
MutationAssessor	3.675	High
FATHMM	-1.4	Tolerable
PROVEAN	-4.81	Damaging
VEST3	0.321	Tolerable
MetaSVM	0.769	Damaging
MetaLR	0.776	Damaging
M-CAP	0.124	Damaging
CADD	23.9	Damaging
DANN	0.994	Damaging
FATHMM MKL	0.993	Damaging
Eigen	0.891	Damaging
GenoCanyon	1.0	Damaging
fitCons	0.487	Tolerable
ReVe	0.691	Tolerable
ClinPred	0.88097769	Pathogenic
REVEL	0.759	Damaging

Fig. S2 The original and unprocessed versions of Fig. 3a.

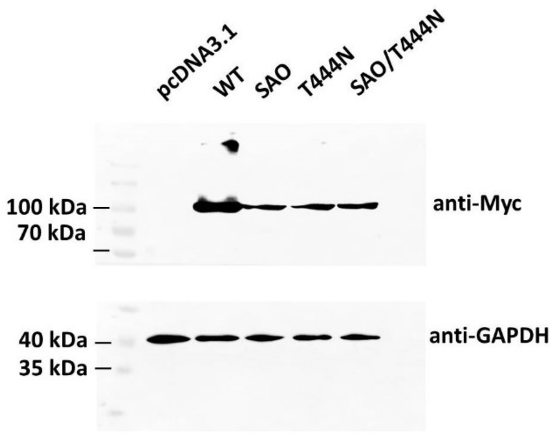

Fig. S3 Cellular localization of wild-type and mutant kAE1 proteins in HEK293T cells detected by immunofluorescence assay. The transfected cells were stained with mouse anti-Myc or rabbit anti-HA antibodies, followed by Hoechst 33342 (blue) and AlexaFluor 488-conjugated anti-mouse (green) or AlexaFluor 594-conjugated anti-rabbit (red) antibodies. The cells were visualized by confocal microscopy. Scale bars: 10 μm.

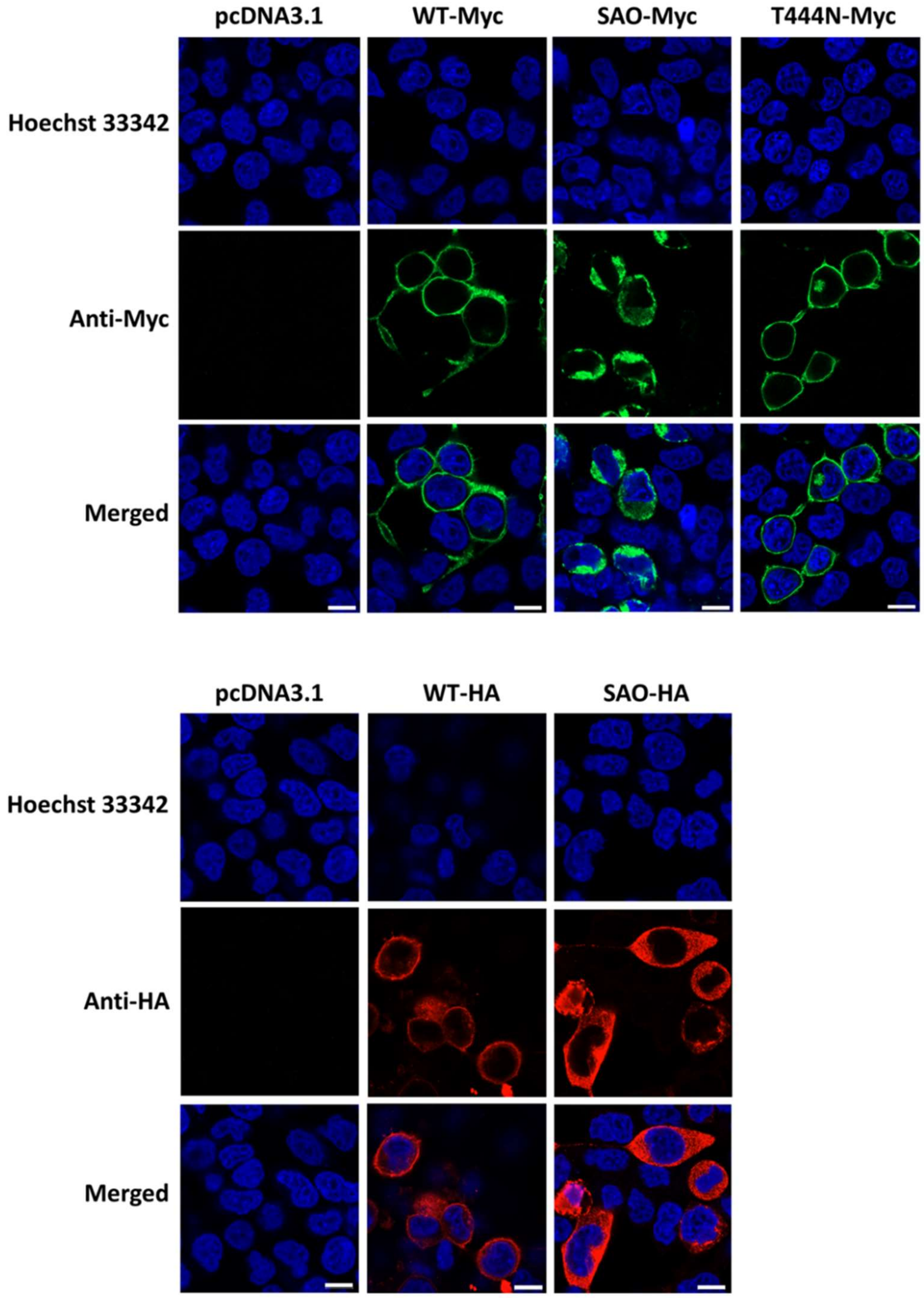

Fig. S4 The original blot and unprocessed versions of Fig. 5a.

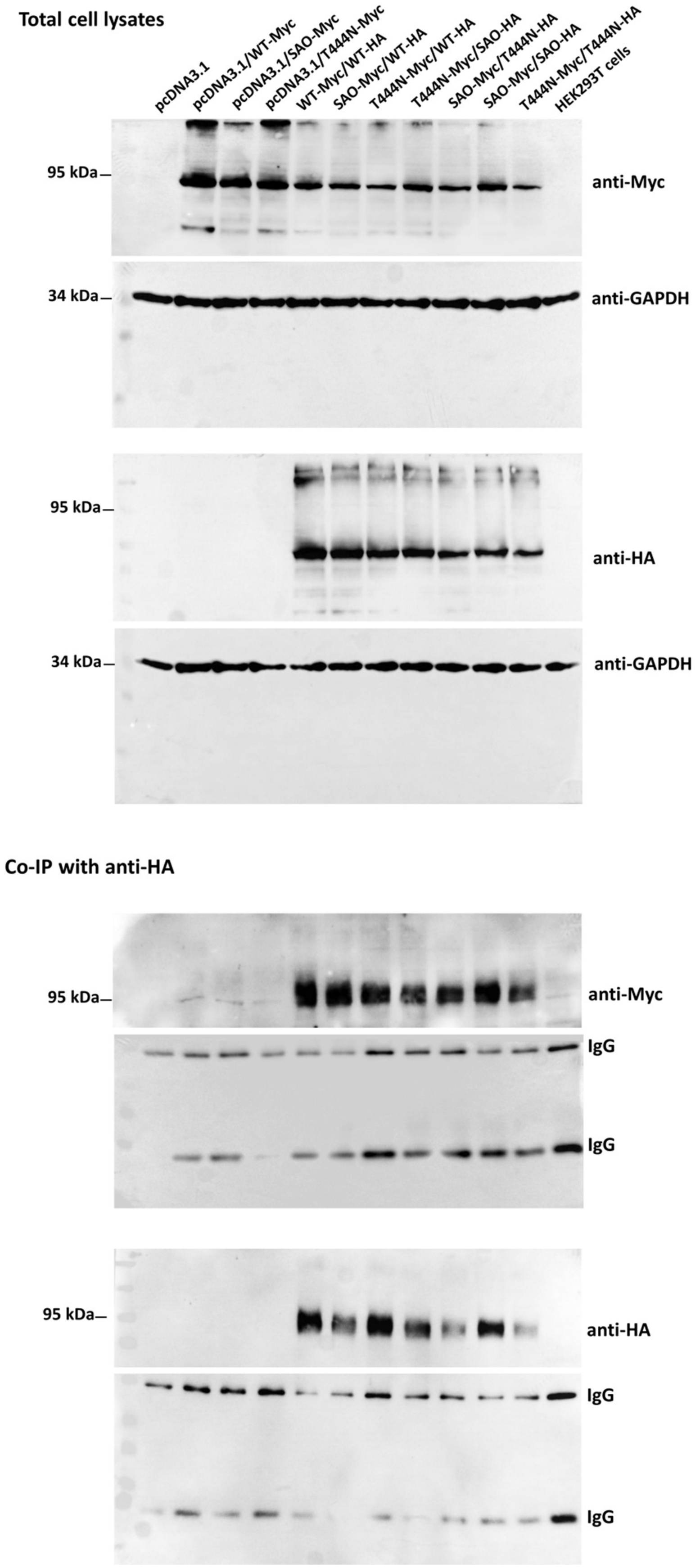

Fig. S5 The original blot and unprocessed versions of Fig. 5b.

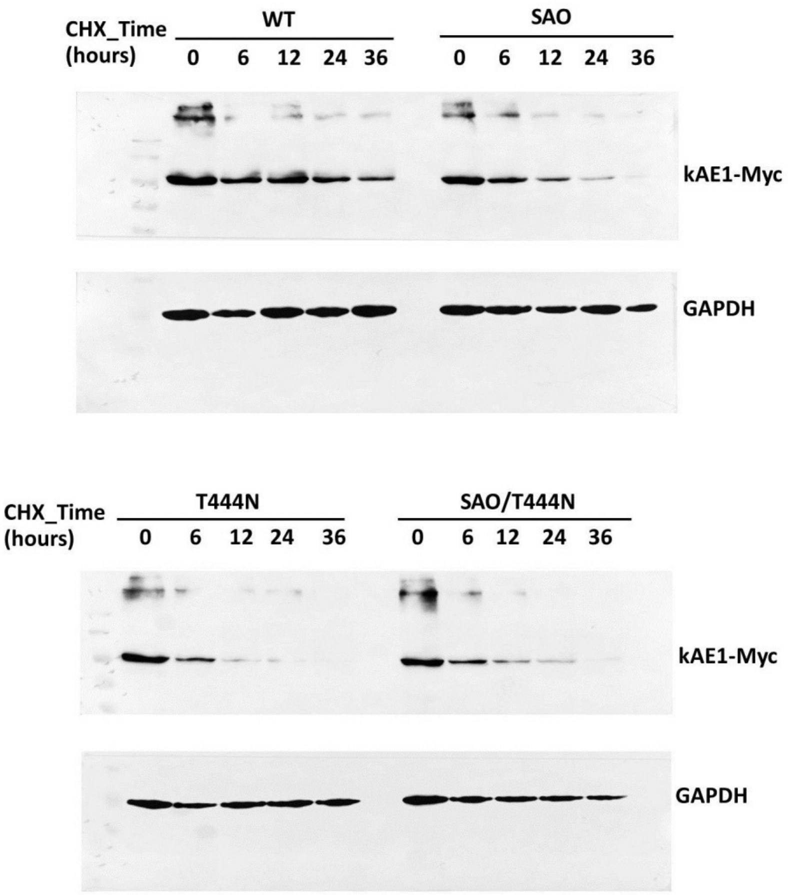

Supplement: Supplementary file 1 — Additional file 1: Table S1: PCR primers for amplification of all exons and exon-intron boundaries of the SLC4A1 gene. Table S2: Clinical and laboratory data of patient (II:1). Table S3: Results of bioinformatic analyses of the T444N mutation using VarCards web-based program. Fig. S1: The original and unprocessed versions of Fig. 1c. Fig. S2: The original and unprocessed versions of Fig. 3a. Fig. S3: Cellular localization of wild-type and mutant kAE1 proteins in HEK293T cells detected by immunofluorescence assay. Fig. S4: The original blot and unprocessed versions of Fig. 5a. Fig. S5: The original blot and unprocessed versions of Fig. 5b. [file 12920_2022_1381_MOESM1_ESM.pdf]
